# Measuring myofascial shear strain in chronic shoulder pain with ultrasound shear strain imaging: a case report

**DOI:** 10.1186/s12891-024-07514-x

**Published:** 2024-05-27

**Authors:** Lingyi Zhao, Jonny Huang, Muyinatu A. Lediju Bell, Preeti Raghavan

**Affiliations:** 1 Department of Electrical and Computer Engineering, Johns Hopkins University, Baltimore, 21218 MD USA; 2 Department of Physical Medicine and Rehabilitation and Neurology, Johns Hopkins Medicine, Baltimore, MD 21287 USA; 3 Department of Biomedical Engineering, Johns Hopkins University, Baltimore, 21218 MD USA; 4 Department of Computer Science, Johns Hopkins University, Baltimore, 21218 MD USA

**Keywords:** Myofascial dysfunction, Shoulder pain, Shear strain, Ultrasound imaging, Case report

## Abstract

**Background:**

Dysfunctional gliding of deep fascia and muscle layers forms the basis of myofascial pain and dysfunction, which can cause chronic shoulder pain. Ultrasound shear strain imaging may offer a non-invasive tool to quantitatively evaluate the extent of muscular dysfunctional gliding and its correlation with pain. This case study is the first to use ultrasound shear strain imaging to report the shear strain between the pectoralis major and minor muscles in shoulders with and without chronic pain.

**Case presentation:**

The shear strain between the pectoralis major and minor muscles during shoulder rotation in a volunteer with chronic shoulder pain was measured with ultrasound shear strain imaging. The results show that the mean ± standard deviation shear strain was 0.40 ± 0.09 on the affected side, compared to 1.09 ± 0.18 on the unaffected side (*p*<0.05). The results suggest that myofascial dysfunction may cause the muscles to adhere together thereby reducing shear strain on the affected side.

**Conclusion:**

Our findings elucidate a potential pathophysiology of myofascial dysfunction in chronic shoulder pain and reveal the potential utility of ultrasound imaging to provide a useful biomarker for shear strain evaluation between the pectoralis major and minor muscles.

## Background

Shoulder pain can have various causes, one of which is myofascial pain and dysfunction [1]. The myofascial system comprises contractile muscle and connective tissue. The connective tissue creates the muscle shape, penetrates the muscle, orients the nerve and vascular endings, and transmits movement from the muscles to the bones to which they are attached [2]. Myofascial pain is characterized by the presence of muscular trigger points (TP), which are hard, palpable nodules located within taut bands in skeletal muscle [3, 4]. Although the cause of myofascial pain is not fully understood, one hypothesis is that any change in the mechano-metabolic environment that produces aggregation of hyaluronan (HA) in the intra- and/or inter-muscular extracellular matrix (ECM) can make the fascia more viscous [2]. This can result in dysfunctional gliding of deep fascia and muscle layers, forming the basis of myofascial pain [5]. Deep or muscular fascia consists of precisely organized, dense, fibrous layers of collagen, elastin, and hyaluronan that interface intricately with the musculature. This enables muscular force to be transmitted efficiently across and between muscles [6].

As a non-invasive and real-time imaging tool, ultrasound (US) imaging can be important in evaluating dysfunctional gliding of muscles [7]. By obtaining real-time visualization of tissue structures and their movements, US imaging can enable clinicians to observe how the muscles and other tissues behave during various movements, which can greatly aid in identifying and assessing dysfunctional gliding.

The most common sonographic findings in patients with myofascial pain are myofascial trigger points, which usually present as hypoechoic nodules within the muscle tissue [8]. In addition, compressing the hypoechoic myofascial trigger point with the US transducer may promptly reproduce the pain (positive sono-palpation). Sonographic mapping of myofascial trigger points can aid accurate and safe intervention techniques such as needling and injection [8].

Combined with dynamic US imaging, shear strain mapping focuses on measuring the deformation or displacement of tissues under mechanical stress or during movement. A previous study has shown that shear strain mapping can quantitatively evaluate the extent of dysfunctional gliding and its correlation with pain [7].

In this case report, we used US shear strain mapping to quantify inter-layer relative motion between the pectoralis major (PMA) and pectoralis minor (PMI) muscles in a volunteer with chronic pain in the left shoulder (i.e., the affected side). We examined the interface between these muscles because tightness in these muscles leads to altered movement of the scapula [9–11]. Furthermore, tightness in the pectoral muscles impedes shoulder internal and external rotation [12–14].

The volunteer performed shoulder external rotation passively using a bimanual arm trainer during which movement of the pectoral muscles was imaged dynamically using ultrasonography. The shear strain between the PMA and PMI muscles was measured and compared between the affected and the unaffected sides.

The remaining contents of this case report are organized as follows: The Case presentation section describes the patient history, experiment setup, measurement methods, and measurement results. The Discussion section contextualizes our key findings, limitations, and future directions. The Conclusions section summarizes the case report.

## Case presentation

### Patient history

The volunteer reported in our study is a 49-year-old woman with chronic pain in her left shoulder for the past two years, reported as 5-6/10 on a numeric rating scale. The pain was exacerbated on arm elevation and lifting heavy objects and was episodic. She noted that the pain was accompanied by tightness in the anterior aspect of the chest. It was relieved with heat, stretching, and occasional use of analgesics (acetaminophen). Physical examination revealed normal range of motion at the shoulder passively. A pain level of 6/10 was elicited on the left shoulder compared to 0/10 on the right shoulder on active shoulder external rotation and elevation. Strength testing in the shoulder muscles was limited by pain.

### Methods

**Fig. 1:**
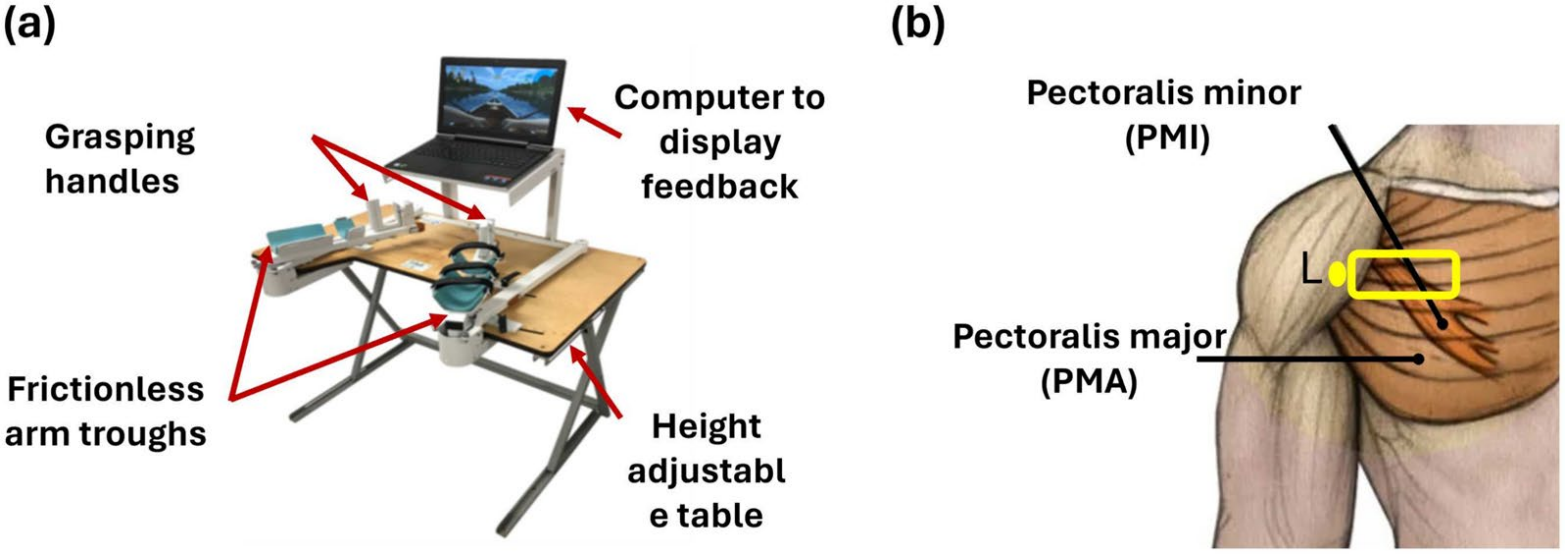
**a** Bimanual arm training device used to move the volunteer’s arms repeatedly. **b** Anatomical locations of the pectoralis minor (PMI) and pectoralis major (PMA) muscles. The yellow rectangle indicates the position of the ultrasound transducer, with ‘L’ denoting the left side of the transducer

We used a bimanual arm training device (Fig. 1a) to repeatedly move the volunteer’s left (affected side) or right (unaffected) arm into shoulder external rotation at a rate of 0.5 Hz. Figure 1b provides a visualization of the anatomical locations of the pectoralis minor (PMI) and pectoralis major (PMA) muscles we examined. We used the bimanual arm trainer because it is an FDA-cleared device designed specifically to provide gentle, controlled passive external rotation at the shoulder, which is especially useful when one shoulder is impaired [15]. The external rotation started from a position of full internal rotation to 30^∘^ of external rotation for each cycle. The arm was abducted to 30^∘^, and the forearm and wrist rested in a neutral position on the arm trough of the device. Passive external rotation in this position using the bimanual arm trainer did not elicit shoulder pain, making the examination relatively comfortable. We acquired a US cine loop (24 Hz) over at least five full rotation cycles using a Clarius L15 scanner (Vancouver, BC, Canada), which is a wireless linear ultrasound transducer with an acoustic frequency range of 5-15 MHz. The transducer was stabilized by a Sawyer robot (Bochum, NRW, Germany) and placed to access the longitudinal plane of the pectoralis muscles. US cine loops of both the affected and unaffected sides were acquired.

Next, we used a cross-correlation-based speckle tracking technique [16] to estimate the inter-frame lateral and axial displacements. Two regions of interest (ROI) with the same lateral location were manually selected on the B-mode image at the beginning of the rotation for movement tracking. The first ROI included the PMI muscle, and the second ROI included the PMA muscle in front of the PMI muscle. The hyperechoic fascial plane was used to delineate the lower boundary of the PMA muscle and the upper boundary of the PMI muscle. For both the affected and unaffected sides, the ROI of the PMA muscle had a size of 6.76 mm $\times$ 2.18 mm, the ROI of the PMI muscle had a size of 6.76 mm $\times$ 2.13 mm, and the distance between the centers of the two ROIs was 2.16 mm. The ROI of the PMA muscle and the PMI muscle will be referred to as PMA ROI and PMI ROI, respectively, for brevity. Although the predominant motion of PMA ROI and PMI ROI during rotation is lateral in the longitudinal view, a small amount of axial tissue motion can also occur. To correct for any axial displacement, we adjusted the inter-frame lateral displacement of the two ROIs based on axial displacement maps. The final inter-frame lateral displacements of the two ROIs were determined after this adjustment. Our displacement estimation approach was tested on simulated and experimental phantom data with known ground truths to ensure the reliability of associated displacement and strain assessments [16, 17].

The average inter-frame displacements associated with the PMA and PMI ROIs were temporally accumulated to obtain the cumulative displacements. The lateral shear strain between the PMI ROI and PMA ROI is defined as:

1 $$\begin{aligned} \text {Shear strain}= \frac{ M_{PMA}-M_{PMI}}{D} \end{aligned}$$

where $M_{PMA}$ is the lateral displacement of PMA ROI, $M_{PMI}$ is the lateral displacement of PMI ROI, and *D* is the distance between the centers of the two ROIs. The sign of the calculated shear strain represents direction rather than magnitude.

To quantitatively compare the shear strain between the affected and unaffected sides, we calculated the shear strain when PMI ROI was at the farthest left of the PMA ROI for each rotation cycle. This moment will be referred to as time P for brevity. The mean shear strain and its standard deviation at time P across all five rotation cycles were calculated to compare the affected and the unaffected sides. The above data analysis was completed with MATLAB software. We ensured reproducibility by executing our algorithm multiple times, verifying that the results were consistent and repeatable across each execution.

### Results

Two videos of PMA and PMI motion during the passive rotation cycles were first recreated from the saved data from one passive rotation cycle in the volunteer’s affected or unaffected shoulder. The PMA ROI was overlaid on the US B-mode image in red, and the PMI ROI in blue The video clips were also customized to show shear strain, cumulative displacement of PMA, and PMI ROI, synchronized with the US B-mode images. The B-mode clips in these two videos show that the PMI and the PMA muscles moved less independently on the affected side compared to the unaffected side. The shear strain and displacement plots demonstrate the same trend, where a larger difference between the PMA and PMI ROI displacement, and thus a larger shear strain, was observed on the unaffected side compared with the affected side.

**Fig. 2:**
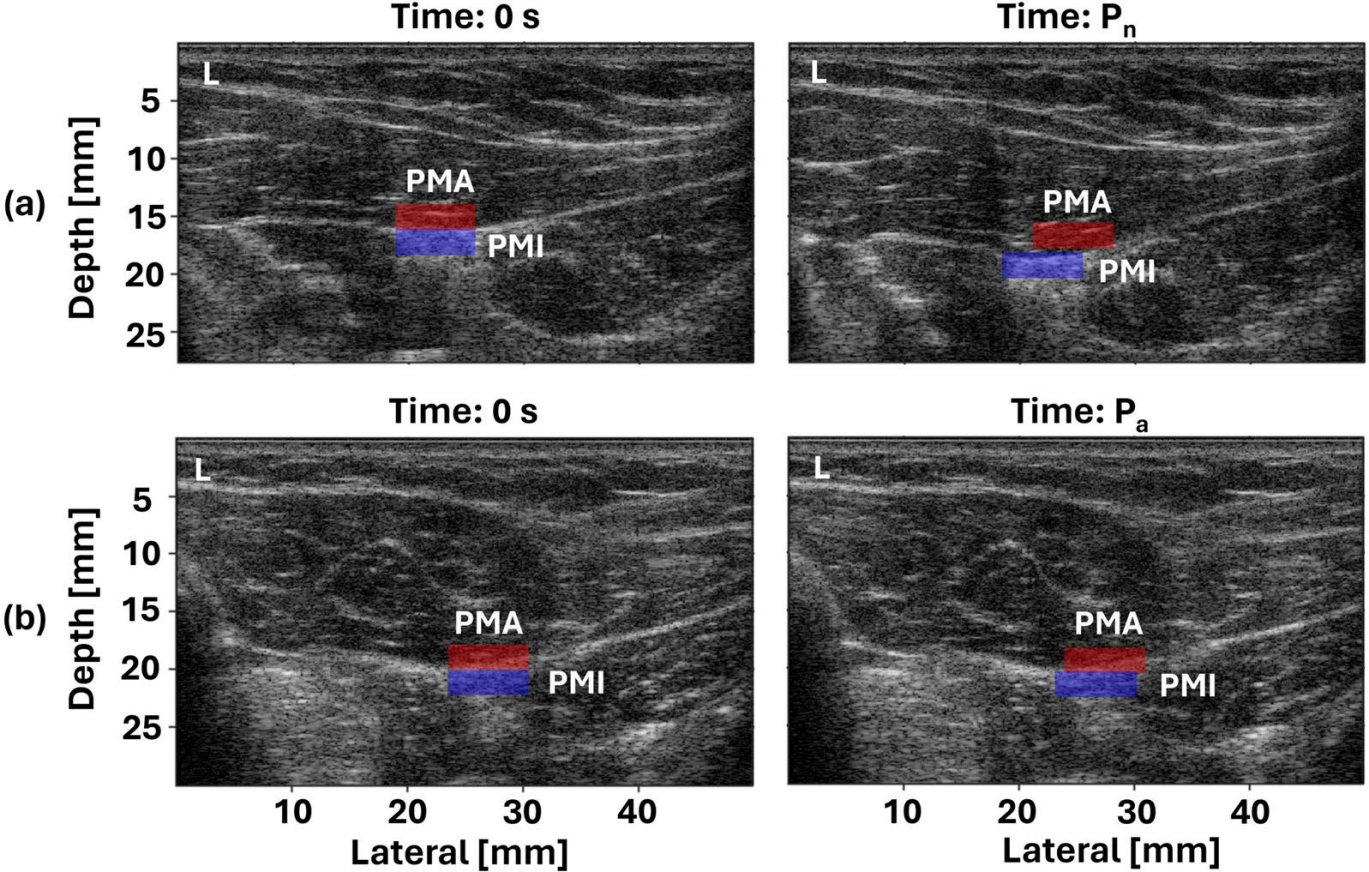
B-mode images with PMA ROI and PMI ROI overlaid in red and blue, respectively, on **a** the unaffected side and **b** the affected side at 0 s, the beginning of the rotation cycle, and at P, when PMI ROI was at the farthest left of PMA ROI. ‘L’ denotes the left side of the transducer

Figure 2 shows B-mode images at two different time points during the first rotation cycle on the unaffected side (Fig. 2a) and the affected side (Fig. 2b). The PMA ROI was overlaid on each B-mode image in red and PMI ROI in blue. Time 0 s represents the beginning of the rotation cycle when PMI ROI and PMA ROI were at the same lateral position. Time $P_{n}$ and time $P_{a}$ represent the moments when PMI ROI was farthest to the left of PMA ROI on the unaffected and affected sides, respectively. In Fig. 2, the difference between the PMA and PMI ROI lateral displacement was larger on the unaffected side at time $P_{n}$ (Fig. 2a) than on the affected side at time $P_{a}$ (Fig. 2b).

**Fig. 3:**
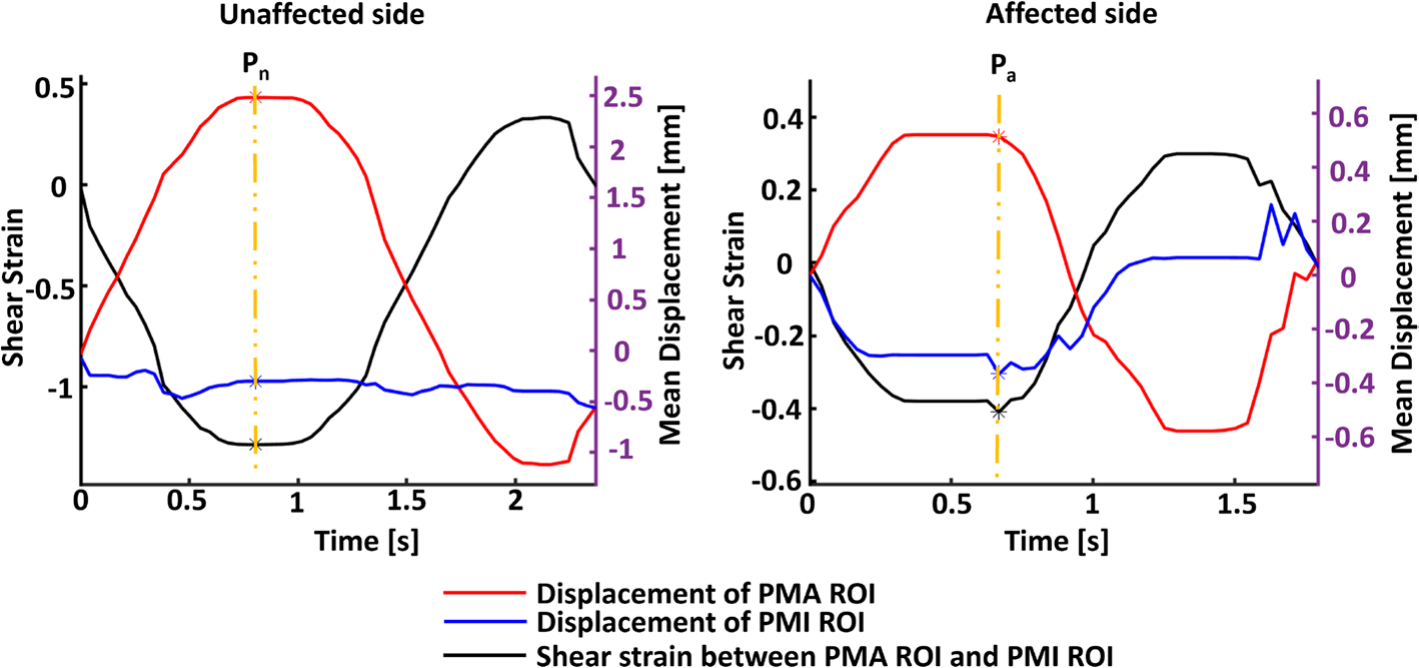
The shear strain and mean displacement of the PMA and PMI ROIs over time during the first rotation cycle on unaffected and affected sides

Figure 3 plots the shear strain between PMI ROI and PMA ROI (left y-axis) and the mean displacement of PMI ROI and PMA ROI (right y-axis) over time in the first rotation cycle. Positive displacement represents movement to the right, and negative displacement represents movement to the left. The vertical dashed lines indicate the time points when the PMI ROI was farthest to the left of the PMA ROI (i.e., $P_{a}$ and $P_{n}$ in Fig. 2). $P_{a}$ and $P_{n}$ also indicate the time points when a single shear strain value was calculated on the affected and unaffected sides for comparison in this rotation cycle.

In Fig. 3, the shear strain at time $P_{a}$ on the affected side was less than the shear strain at time $P_{n}$ on the unaffected side. The same trend was observed when calculating the mean ± standard deviation shear strain at time P across all five rotation cycles. Specifically, the mean ± standard deviation shear strain at time P across five rotation cycles on the affected side was 0.40 ± 0.09, significantly lower than the mean ± standard deviation shear strain on the unaffected side at 1.09 ± 0.18 (*p*<0.05).

## Discussion

This case study is the first to evaluate the shear strain between the PMA and PMI muscles of the affected and the unaffected sides of a human subject with chronic shoulder pain using dynamic US imaging. Previous reports have investigated the relationship between the shear strain within the thoracolumbar fascia and lower back pain, demonstrating that thoracolumbar fascia shear strain was reduced in human subjects with lower back pain compared to those without lower back pain [7]. Our case study extends the shear strain evaluation method to the PMI and PMA muscles. In alignment with the reports in [7], our results demonstrate that the shear strain between the PMA muscle and the PMI muscle on the affected side is less than that on the unaffected side (i.e., $0.40\pm 0.09$ vs $1.09\pm 0.18$). These results suggest that shear strain between the PMA muscle and the PMI muscle may be able to serve as a biomarker to elucidate the pathophysiology of myofascial dysfunction in the shoulder and potentially aid in monitoring the progression or response to treatment in individuals with myofascial dysfunction in the shoulder. Note that our analysis was designed to compare the shear strain between the affected and the unaffected side in a single case and does not aim to infer statistical significance across a broader population.

One possible explanation for the observed reduced shear strain on the affected side is the aggregation of HA in the muscle and its fascia. In physiologic quantities, HA functions as a lubricant and as a viscoelastic shock absorber [18]. Previous work has suggested that the aggregation of HA can result in increased tissue viscosity, which may lead to the formation of the taut bands that constitute trigger points, causing dysfunctional gliding of intra- and intermuscular deep fascial planes [19]; this may result in a lower shear strain.

The sonographic features of the deep fascia include multiple hyperechoic (fibrous layers) and hypoechoic (loose connective tissue) lines parallel to each other. Differential gliding of the fibrous layers is expected in healthy muscle during movement as measured by the shear strain. In contrast, HA aggregation may lead to thickening of the deep fascia and/or adhesions between the layers of connective tissue [6, 20], consequently reducing the shear strain.

To further validate our findings, future work will enroll more patients. In addition, the accuracy of identifying the appropriate imaging plane for evaluation may vary depending on the operator’s skills. Artificial intelligence-navigated US imaging may be investigated to aid in image plane-finding to enhance the accuracy and efficiency of the evaluation process [21, 22]. Additional future work will include investigating the relationship among shear strain, myofascial pain, and HA aggregation, which will verify the role of shear strain as a biomarker for myofascial dysfunction and pain.

## Conclusions

This case study is the first to report the shear strain between the PMA and PMI muscles in a human subject with chronic shoulder pain. The shear strain between the PMA and PMI muscles on the affected side was reduced compared to the unaffected side. Our findings are promising for using US imaging for shear strain evaluation between the PMA and PMI muscles, which may serve as a useful biomarker to elucidate the pathophysiology of myofascial dysfunction.

## Data Availability

The datasets used and analyzed during the current study are available from the corresponding authors upon reasonable request.
